# Fungal Infection following Total Elbow Arthroplasty

**DOI:** 10.1155/2019/7927914

**Published:** 2019-09-04

**Authors:** Samuel S. Ornell, Khang H. Dang, Aaron J. Bois, Anil K. Dutta

**Affiliations:** ^1^The University of Texas Health Science Center at San Antonio, Department of Orthopaedics, San Antonio, TX 78229, USA; ^2^Section of Orthopaedic Surgery, Department of Surgery, University of Calgary, Calgary, AB, Canada

## Abstract

A specific treatment protocol for managing fungal infections after total elbow arthroplasty (TEA) does not currently exist. The purpose of this report is to describe our experience and outline our treatment algorithm for a rare case of prosthetic joint infection (PJI) following a TEA. We present a case of a PJI due to *Candida parapsilosis* after TEA in a 57 year-old Caucasian woman with a history of hypertension, depression, and three previous surgical procedures to the affected limb. A fungal PJI by the organism *C. parapsilosis* following TEA has not been previously reported. Successful eradication of the fungal infection was achieved utilizing resection arthroplasty; placement of an amphotericin, vancomycin, and tobramycin-impregnated cement spacer; and 6 months of organism-specific antifungal medication. Although the patient was clinically ready for reimplantation, she passed away due to unrelated issues before reimplantation could be performed. While PJI is a devastating complication following TEA, a fungal infection is a rare complication that imposes difficult challenges to the treating surgeon. With our case report, we hope to contribute to the overall knowledge of fungal infections associated with TEA and describe our successful treatment of this complex case.

## 1. Introduction

Advances in surgical technique along with innovations in implant design have allowed total elbow arthroplasty (TEA) to become an increasingly common and effective treatment option for patients with rheumatoid arthritis, persistent joint instability, unsalvageable distal humerus fractures, and end-stage osteoarthritis [1–5]. While TEA has shown to improve pain, functional outcomes, and quality of life, complication rates remain high (20% to 45%) [1, 2]. The most common complications include implant loosening and/ or failure, instability (i.e., dislocation or subluxation), injury to the ulnar nerve, intraoperative fracture, and heterotopic ossification [2, 3].

The rate of prosthetic joint infection (PJI) in TEA has been reported to be 5% to 8%, a rate higher to that of both total knee and total hip arthroplasty [1–3]. The most common causes of infection are bacterial, namely, Staphylococcus epidermidis [5]. Here, we present a case of a fungal PJI due to *Candida parapsilosis* following a TEA in a 57 year-old Caucasian woman. To our knowledge, only one other case of fungal PJI after TEA has been reported in the orthopaedic literature; however, a case of fungal PJI by the organism *C. parapsilosis* has not been previously reported.

To date, a specific treatment protocol for managing fungal infections following TEA does not exist. The purpose of this report is to describe our experience and outline our treatment algorithm for this rare cause of PJI following a TEA.

## 2. Case Report

A 57 year-old, right hand-dominant female sustained a right distal humerus fracture from a fall in mid-2009; her past medical history was significant for hypertension and depression. The patient underwent fracture fixation and collateral ligament repair at an outside institution. Due to persistent elbow pain and instability, revision collateral ligament repair was performed in late 2009. In early 2010, the patient underwent lateral collateral ligament reconstruction utilizing allograft tissue by her initial surgeon due to reported recurrent elbow instability.

**Figure 1:**
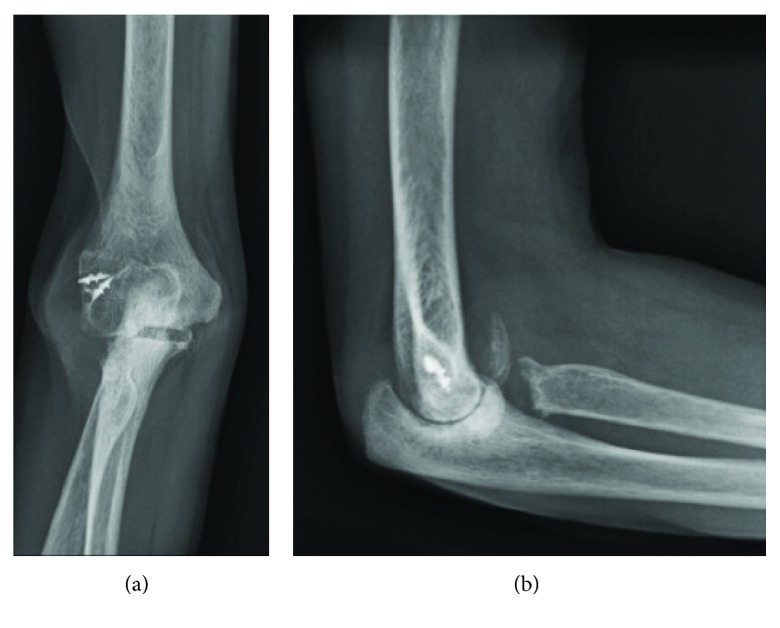
(a) AP and (b) lateral radiographs of the right elbow obtained upon the initial presentation to our clinic.

**Figure 2:**
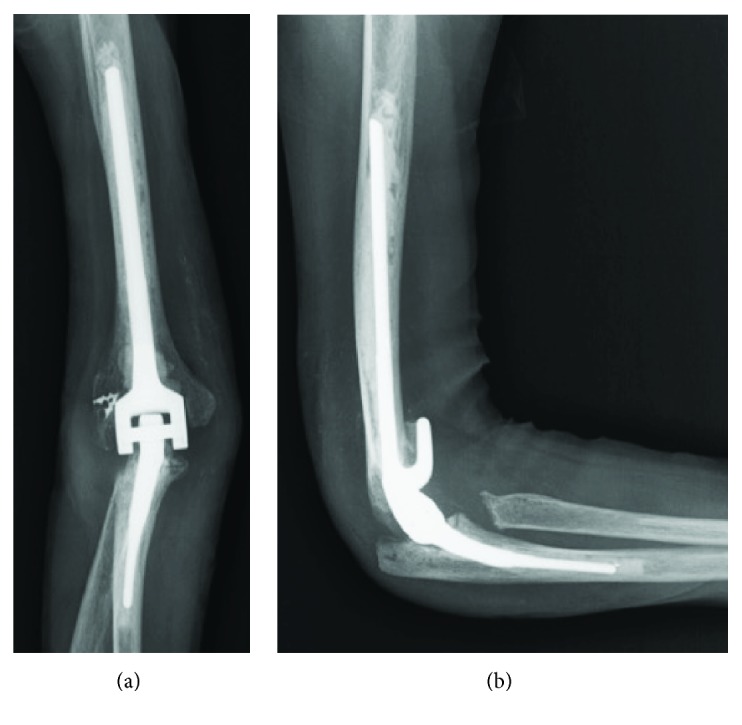
(a) AP and (b) lateral radiographs of the right elbow following a long-stemmed cemented Coonrad-Morrey TEA.

**Figure 3:**
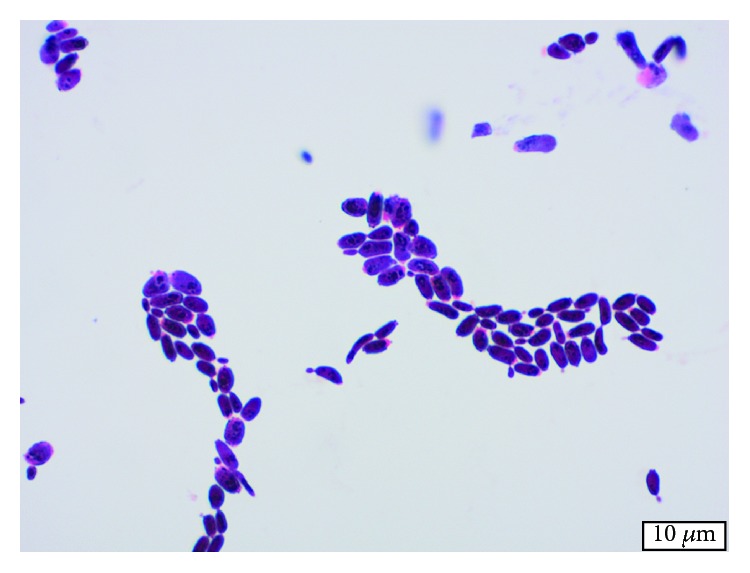
Gram stain of *Candida parapsilosis* at 1000x magnification.

**Figure 4:**
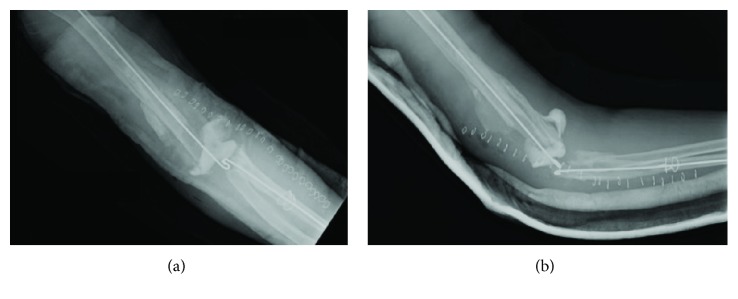
(a) AP and (b) lateral radiographs obtained following resection arthroplasty with placement of an amphotericin, vancomycin, and tobramycin-impregnated cement spacer.

**Figure 5:**
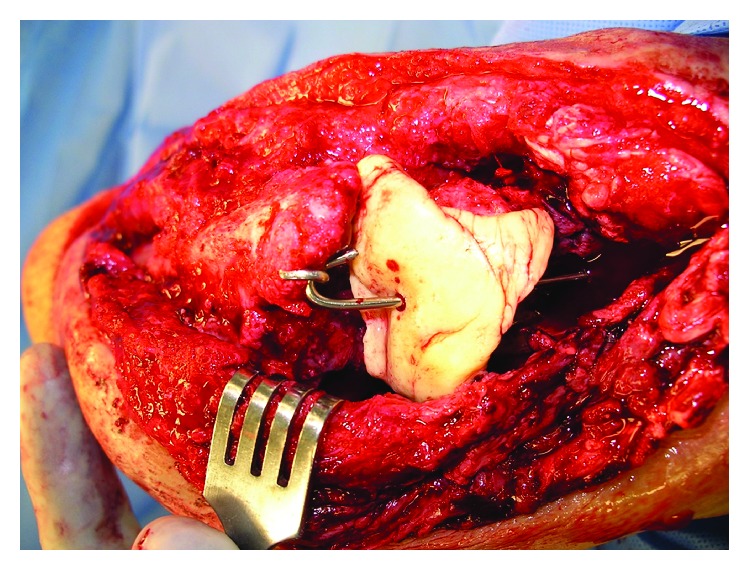
Intraoperative photograph of the implanted amphotericin, vancomycin, and tobramycin-impregnated cement spacer.

Nine months after the ligament reconstruction procedure, the patient presented to our orthopaedic department with severe right elbow pain and instability (Figure 1). She reported no specific history of recent trauma or infectious symptoms such as fever, chills, or other sites of infection. Given her age and functional status, the options of conservative management, interposition arthroplasty, or total elbow arthroplasty were discussed. Preoperative blood work consisting of a complete blood count (CBC), erythrocyte sedimentation rate (ESR), and C-reactive protein (CRP) serum laboratory values was obtained and within a normal range. The patient proceeded with a long-stemmed cemented Coonrad-Morrey TEA (Figure 2). Approximately 6 weeks later, in mid-2011, she developed a painless, persistent draining sinus from the posterior aspect of the elbow. The patient strongly desired to retain the elbow replacement and was agreeable to undergo a debridement procedure. Intraoperative cultures grew *Candida parapsilosis*, and she was subsequently placed on chronic fluconazole (Figure 3). However, in late 2011, she required a resection arthroplasty after multiple debridements, a bushing exchange, and antimicrobial regimens failed to resolve the persistent draining sinus. At the time of resection, there was no evidence of implant loosening. The implant was replaced with an amphotericin, vancomycin, and tobramycin-impregnated cement spacer (Figures 4 and 5). She also underwent fixation of an ulnar fracture sustained from a fall prior to undergoing the resection arthroplasty procedure. Intraoperative cultures at this time grew Coagulase-negative staphylococcus and methicillin-sensitive staphylococcus aureus. She was treated as a mixed fungal and bacterial infection and treated with 6 months of fluconazole and 6 weeks of vancomycin. In the following 6 months, she reported no signs of infection such as erythema, soft tissue fluctuance, or drainage, and her neurovascular exam was unremarkable other than decreased ulnar nerve sensation. She was clinically ready for reimplantation in late 2012. Unfortunately, the patient died due to unrelated circumstances before reimplantation could be performed.

## 3. Discussion

Total elbow arthroplasty has experienced numerous advancements in both implant design and surgical technique. Despite these efforts to improve outcomes, the rate of complications remains higher than that of both total knee and hip arthroplasty, with reported complication rates as high as 45% [1, 2]. After aseptic loosening and instability, PJI is the third most common complication following TEA, with a reported rate of 5% to 8% [1–3].

We present a previously unreported case of fungal PJI with the organism *C. parapsilosis* following a TEA. Most PJIs, including those involving the shoulder, hip and knee, are caused by the organisms *Staphylococcus aureus* and *S epidermidis*, whereas fungal organisms represent a mere 1% of the etiologies, with the Candida species being the most common [1, 3, 5–10]. The decision to perform an initial surgical debridement, bushing exchange, and implant retention was based on the well-fixed implants, minimal patient symptoms, and the concern for bone loss in the setting of elbow explantation [11]. Our organism, *C. parapsilosis*, similar to *C. albicans* and *S. epidermidis*, produces a biofilm which increases its virulence and promotes a greater resistance to antifungal medications. Overall, these attributes create a technically challenging infection for the treating surgeon [1, 7, 8].

Risk factors associated with PJI include medical comorbidities, such as rheumatologic disease, diabetes mellitus, renal insufficiency, and immunocompromised states, such as transplant patients, human immunodeficiency virus (HIV), chronic corticosteroid use, and chronic antibiotic use [2, 7, 8]. Specific risk factors for candidal infection include intravenous drug use, broad spectrum antibiotics, indwelling catheters, immunosuppression, severe burns, and recent surgery [6–8]. Azzam et al. [7] also reported that multiple revision surgeries or complex reconstructions with prolonged hospitalization may increase the risk of a candida infection. Our patient did have multiple revision surgeries and a complex reconstruction. In addition, the inherent characteristics of a TEA may also lead to increased rates of infection. The elbow offers limited soft tissue coverage for the implant and is subject to tensile forces during range of motion, which can lead to wound dehiscence [2, 3].

To the best of our knowledge, only one other case of fungal infection following a TEA exists in the literature. Kwong et al. [1] reported a patient with rheumatoid arthritis who experienced a PJI by the organism *Aspergillus terreus*. Initially, the patient required a resection arthroplasty due to a persistent draining sinus and a coagulase-negative staphylococcus infection. After staged treatment with a vancomycin-impregnated cement spacer and 6 weeks of ciprofloxacin and vancomycin for a simultaneous *Enterobacter cloacae* infection, the patient underwent subsequent TEA reimplantation but developed clinical signs of infection at six months postoperatively. The joint aspiration grew *Aspergillus terreus*, which was treated with 8 weeks of intravenous voriconazole and vancomycin. Unfortunately, while receiving immunomodulators for a rheumatoid flare, symptoms recurred requiring a repeat resection arthroplasty and course of antifungal medications. For patients such as this with immune-mediated disease undergoing TEA, limitations exist on the interpretation of inflammatory markers such as ESR and CRP. Such patients can have elevated basal levels of ESR or CRP that have the potential to cause false-positive tests, and the use of immunosuppressive therapies such as disease-modifying antirheumatic drugs (DMARDs) can also affect the accuracy of test results [12].

Due to the rarity of fungal infection in TEA, specific treatment guidelines for this pathology do not exist. Currently, the Infectious Disease Society of America (IDSA) recommends against sole treatment with systemic antifungal agents or one-stage exchange arthroplasty in fungal PJI in TKA and THA [4]. Instead, a two-stage exchange arthroplasty is recommended, along with organism-specific intravenous or highly bioavailable oral antimicrobial therapy for 4 to 6 weeks [4]. The current literature supports that a two-stage revision arthroplasty provides the highest likelihood of successful reimplantation in cases of PJI following TEA [1, 3, 6, 7, 9, 13]. At the time of reimplantation, multiple tissue biopsies should be obtained for culture, with the addition of fungal-selective cultures [9, 12, 14]. Additional options include antibiotic eluting spacers, with the benefit of direct localization of antimicrobial agents to the infected space; however, few studies have investigated the efficacy of these spacers in the setting of fungal infection. While some agree that these spacers aid in preventing or treating a superimposed bacterial infection in the setting of fungal PJI, the efficacy of eradicating a fungal infection by mixing antifungal agents within the cement remains unknown due to minimal rates of drug elution [6, 7]. We note that the placement of an amphotericin, vancomycin, and tobramycin-impregnated cement spacer was part of our successful treatment.

While a PJI remains a devastating complication following TEA, a fungal PJI imposes additional challenges to the treating surgeon. Patients with multiple medical comorbidities, an immunocompromised state, and a history of multiple elbow procedures may be at an increased risk of developing a fungal infection. Treatment guidelines for the management of fungal infection after TEA currently do not exist. Here, we describe the successful treatment of a *C. parapsilosis* fungal PJI after TEA utilizing resection arthroplasty, placement of an amphotericin, vancomycin, and tobramycin-impregnated cement spacer and 6 months of organism-specific antifungal medication. With our case report, we hope to contribute to the overall knowledge of fungal infections associated with TEA and describe our successful treatment of this complex case.

## References

[1] Kwong C. A., Puloski S. K. T., Hildebrand K. A. (2017). Fungal periprosthetic joint infection following total elbow arthroplasty: a case report and review of the literature. *Journal of Medical Case Reports*.

[2] Voloshin I., Schippert D. W., Kakar S., Kaye E. K., Morrey B. F. (2011). Complications of total elbow replacement: a systematic review. *Journal of Shoulder and Elbow Surgery*.

[3] Kim J. M., Mudgal C. S., Konopka J. F., Jupiter J. B. (2011). Complications of total elbow arthroplasty. *The Journal of the American Academy of Orthopaedic Surgeons*.

[4] Osmon D. R., Berbari E. F., Berendt A. R. (2012). Diagnosis and management of prosthetic joint infection: clinical practice guidelines by the Infectious Diseases Society of America. *Clinical Infectious Diseases*.

[5] Yamaguchi K., Adams R. A., Morrey B. F. (1998). Infection after total elbow arthroplasty. *The Journal of Bone and Joint Surgery*.

[6] Kuiper J. W. P., van den Bekerom M. P. J., van der Stappen J., Nolte P. A., Colen S. (2013). 2-stage revision recommended for treatment of fungal hip and knee prosthetic joint infections. *Acta Orthopaedica*.

[7] Azzam K., Parvizi J., Jungkind D. (2009). Microbiological, clinical, and surgical features of fungal prosthetic joint infections: a multi-institutional experience. *The Journal of Bone and Joint Surgery. American Volume*.

[8] Bariteau J. T., Waryasz G. R., McDonnell M., Fischer S. A., Hayda C. O. L. R. A., Born C. T. (2014). Fungal osteomyelitis and septic arthritis. *The Journal of the American Academy of Orthopaedic Surgeons*.

[9] Gebauer M., Frommelt L., Achan P. (2014). Management of fungal or atypical periprosthetic joint infections. *The Journal of Arthroplasty*.

[10] Koutserimpas C., Zervakis S. G., Maraki S. (2019). Non-*albicans Candida* prosthetic joint infections: a systematic review of treatment. *World Journal of Clinical Cases*.

[11] Streubel P. N., Simone J. P., Morrey B. F., Sanchez-Sotelo J., Morrey M. E. (2016). Infection in total elbow arthroplasty with stable components: outcomes of a staged surgical protocol with retention of the components. *The Bone & Joint Journal*.

[12] Yeganeh M. H., Kheir M. M., Shahi A., Parvizi J. (2018). Rheumatoid arthritis, disease modifying agents, and periprosthetic joint infection: what does a joint surgeon need to know?. *The Journal of Arthroplasty*.

[13] Cheung E. V., Adams R. A., Morrey B. F. (2008). Reimplantation of a total elbow prosthesis following resection arthroplasty for infection. *The Journal of Bone and Joint Surgery. American Volume*.

[14] Ahmadi S., Lawrence T. M., Morrey B. F., Sanchez-Sotelo J. (2013). The value of intraoperative histology in predicting infection in patients undergoing revision elbow arthroplasty. *The Journal of Bone and Joint Surgery. American Volume*.

